# Synergistic interactions between phenolic compounds identified in grape pomace extract with antibiotics of different classes against *Staphylococcus aureus* and *Escherichia coli*

**DOI:** 10.1371/journal.pone.0172273

**Published:** 2017-02-24

**Authors:** Loreto Sanhueza, Ricardo Melo, Ruth Montero, Kevin Maisey, Leonora Mendoza, Marcela Wilkens

**Affiliations:** 1 Núcleo de Química y Bioquímica, Facultad de Ciencias, Universidad Mayor, Santiago, Chile; 2 Laboratorio de Inmunología Comparativa, Departamento de Biología, Facultad de Química y Biología, Universidad de Santiago de Chile, Santiago, Chile; 3 Laboratorio de Micología, Departamento de Química de los Materiales, Facultad de Química y Biología, Universidad de Santiago de Chile, Santiago, Chile; 4 Laboratorio de Microbiología Básica y Aplicada, Departamento de Biología, Facultad de Química y Biología, Universidad de Santiago de Chile, Santiago, Chile; Institute of medical research and medicinal plant studies, CAMEROON

## Abstract

Synergy could be an effective strategy to potentiate and recover antibiotics nowadays useless in clinical treatments against multi-resistant bacteria. In this study, synergic interactions between antibiotics and grape pomace extract that contains high concentration of phenolic compounds were evaluated by the checkerboard method in clinical isolates of *Staphylococcus aureus* and *Escherichia coli*. To define which component of the extract is responsible for the synergic effect, phenolic compounds were identified by RP-HPLC and their relative abundance was determined. Combinations of extract with pure compounds identified there in were also evaluated. Results showed that the grape pomace extract combined with representatives of different classes of antibiotics as β-lactam, quinolone, fluoroquinolone, tetracycline and amphenicol act in synergy in all *S*. *aureus* and *E*. *coli* strains tested with FICI values varying from 0.031 to 0.155. The minimal inhibitory concentration (MIC) was reduced 4 to 75 times. The most abundant phenolic compounds identified in the extract were quercetin, gallic acid, protocatechuic acid and luteolin with relative abundance of 26.3, 24.4, 16.7 and 11.4%, respectively. All combinations of the extract with the components also showed synergy with FICI values varying from 0.031 to 0.5 and MIC reductions of 4 to 125 times with both bacteria strains. The relative abundance of phenolic compounds has no correlation with the obtained synergic effect, suggesting that the mechanism by which the synergic effect occurs is by a multi-objective action. It was also shown that combinations of grape pomace extract with antibiotics are not toxic for the HeLa cell line at concentrations in which the synergistic effect was observed (47 μg/mL of extract and 0.6–375 μg/mL antibiotics). Therefore, these combinations are good candidates for testing in animal models in order to enhance the effect of antibiotics of different classes and thus restore the currently unused clinical antibiotics due to the phenomenon of resistance. Moreover, the use of grape pomace is a good and low-cost alternative for this purpose being a waste residue of the wine industry.

## Introduction

Antibiotics are of immense value for fighting bacterial infections, however, their effectiveness has been threatened by the continuing emergence of bacteria resistant to these drugs [1], becoming the main cause of failure in the treatment of infectious diseases [2]. Currently, there are more than 15 kinds of antibiotics whose action sites are related to physiological or metabolic functions essential for the bacteria. Unfortunately, none have escaped the resistance phenomenon [3], increasing the number of pathogenic bacteria that show a phenotype of resistance to multiple antibiotics (MDR), as for example methicillin resistant *Staphylococcus aureus* (MRSA) and *Escherichia coli* clinical isolates [1, 4]. It is for these reasons that new alternatives need to be sought. One strategy dealing this problem is the synergy using combinations of natural compounds with antibiotics and thus enhancing or restoring the antibacterial activity of many antibiotics currently useless because of bacterial resistance mechanisms acquisition.

Different combinations may improve or facilitate the interaction of an antibiotic with its target inside the bacterial cell, and in addition, some compounds should act by a different mechanism as the known antibacterial agent. The synergy can be used to expand the antimicrobial spectrum, prevent the emergence of antibiotic resistant bacteria, and to minimize toxicity, since lower concentrations of both agents can be used. Many *in vitro* studies have been published which show the synergistic effect between plant extracts with antibiotics of different classes against sensitive and multi-drug resistant pathogenic strains. Betoni et al. [5] showed that plants possess antibacterial compounds that may act in synergy by sensitizing the pathogen to the antibiotic. Moreover, several studies have found that the combination of antimicrobial agents with plant extracts reduced the minimum inhibitory concentration of antibiotics in different MDR bacteria [6–8].

A rich resource of phenolic compounds is the grape pomace (*Vitis vinifera*), which is the main organic waste generated in the industries of wine [9] representing between 13–20% of the total weight of the processed grapes [10]. The phenolic compounds content in pomace includes phenolic acids (gallic, sinapic, protocatechuic, etc.), and flavonoids as flavan-3-ol such as (+)-catechin, (-)-epicatechin, (-)-epicatechin-3-O-gallate, quercetin, miricetin, kaempferol, luteolin, among others [11, 12].

The antibacterial activity of the phenolic compounds identified in grape has been studied in extracts obtained from fruits [13], seeds [14–16], skin [17] and pomace [15, 18, 19]. Moreover, it has been determined that phenolic compounds act in synergy with different classes of antibiotics [20–22]. Some examples are epicatechin gallate (EGC) and epigallocatechin gallate (EGCg), able to reduce 64 times the MIC of oxacillin MRSA strains [23], as well as baicalein with tetracyclines and β-lactamics [24]. Furthermore, curcumin acts in synergy with β-lactamics reducing the MIC of oxacillin and ampicillin16 times, and 25 times the MIC of ciprofloxacin [25]. Within the mechanisms by which the phenolic compounds exert their antibacterial effect, it is described that are capable of interacting with the cytoplasmic membrane, cell wall, nucleic acids and/or energy transport [2], altering or inhibiting their functions.

In this study, the synergy between grape pomace extracts with antibiotics from different classes was determined against multi-resistant clinical isolates of *S*. *aureus* and *E*. *coli*. In addition, it was evaluated if pure phenolic compounds present in grape pomace extracts exert this synergistic effect and cytotoxicity determination of synergic combinations was also included.

## Materials and methods

### Grape pomace extract

The grape pomace of Cabernet Sauvignon variety was obtained from Viña Miguel Torres (Curicó, Chile). Samples (900 g) were ground and extracted with methanol/HCl 1% (v/v) for 18 h at 4°C under constant agitation (100 rpm). Samples were concentrated on a rotary evaporator at 50°C and subjected to liquid-liquid extraction with ethyl acetate [12]. Finally, the samples were concentrated to dryness and kept at -20°C.

### Compounds identification by chromatographic analysis

Phenolic compounds present in the grape pomace extract were separated through RP-HPLC as described by Mendoza et al. [12] using a Waters 600 HPLC chromatograph (Waters, Mildford, MA, USA) equipped with a Waters 2990 diode detector and a C-18 column (3,9 mm x 150 mm; Waters, Mildford, MA, USA). A gradient program consisting of solvent A (acetic acid 1% (v/v) in distilled water) and solvent B (acetonitrile 100%) was applied at a flow rate of 0.8 mL/min as follows: a linear gradient of 10 to 20% B in 20 min; this proportion was maintained for the following 20 min; and then, a linear gradient from 20 to 50% B in 5 min; this last proportion was maintained up to 60 min. Grape pomace extract and individual phenolic compounds were prepared at 5 mg/mL in 1 mL methanol and 20 μL of sample solution was injected. The detector was set at 280 and 360 nm. Identification of phenolic compounds was done by comparison of UV—vis spectra and retention times with standards using Millenium 3.20 software. All standards, gallic acid, protocatechuic acid, syringic acid, vanillic acid, p-coumaric acid, ellagic acid, kaempferol, quercetin, luteolin, (+)-catechin and (-)-epicatechin, were obtained from Sigma Aldrich. Each sample was analyzed in triplicate.

### Bacterial strains and culture media

Five clinical isolates of *S*. *aureus*, strains 8298–2, 8275, and methicillin-resistant *S*. *aureus* (MRSA) strains 622–4, and 97–7, kindly donated by Dr. Gino Corsini (Universidad Autónoma, Chile) were used; and as a control strain, *S*. *aureus* ATCC 6538. For *E*. *coli*, three clinical isolates (33.1, 16.1 and A2UC, kindly donated by the Instituto de Salud Pública de Chile; and the control *E*. *coli* ATCC 25922 were used. All strains were cultured on Luria-Bertani (LB) agar and incubated at 37°C for 18–24 h.

### Antibiotic sensitivity assays

Antibiotic susceptibility of all bacterial strains was determined following the disc agar diffusion assay described by Clinical Laboratory Standards (CLSI) [26]. The bacterial strains were cultured overnight, diluted in Mueller Hinton (MH) broth to a McFarland turbidity of 0.5 (1 x 10^8^ colony forming unit (CFU)/mL) and seeded homogeneously on Petri dishes containing Mueller Hinton agar. Sterile discs containing different concentrations of antibiotics were placed on the inoculated agar. After incubation for 18 h at 37°C, the inhibition diameters were measured and these values in millimeter (mm) were interpreted according to the criteria established by CLSI as resistant (R) or sensitive (S). Antibiotics used for *S*. *aureus* susceptibility determination were nalidixic acid (30 μg), oxacillin (1 μg), ciprofloxacin (5 μg), norfloxacin (10 μg), levofloxacin (5 μg) tetracycline (30 μg), and chloramphenicol (30 μg). The same antibiotics were used for *E*. *coli*, except that the oxacillin was replaced with ampicillin (10 μg).

### Minimal inhibitory concentration determination

Minimal inhibitory concentration (MIC) of four different classes of antibiotics against all used strains was determined. As representative of the β-lactam class, oxacillin and ampicillin; nalidixic acid for quinolone; the fluoroquinolones class represented by ciprofloxacin, norfloxacin and levofloxacin; tetracycline representing tetracycline family; and finally, chloramphenicol as representative of the antibiotic class amphenicol. All these drugs were obtained from Sigma Aldrich. The MIC was also determined for grape pomace extract and the following phenolic compounds identified in pomace in this work as gallic acid, syringic acid, vanillic acid, quercetin, kaempferol, luteolin, (+)-catechin, and (-)-epicatechin.

For all the above-mentioned drugs, compounds and extract, the MIC determination assay was followed as established by CLSI using the micro-broth dilutions method in 96-well plates (Nunc) at different concentration ranges. To each well, 188 μL of MH broth, 10 μL of pomace extract (300 to 3,000 μg/mL diluted in dimethyl sulfoxide (DMSO)), antibiotic (0.75 to 3,000 μg/mL) or pure phenolic compound (200 to 10,000 μg/mL diluted in DMSO), and finally 2 μL bacterial suspension at McFarland 0.5 (1 x 10^8^ CFU/mL) to complete a final volume of 200 μL. In addition, some wells were used as solvent and sterility controls. The plates were incubated at 37°C for 24 h and the optical density was measured at 600 nm in an Elisa lector (Thermos Labsystems Multiskan FC Model). Results are expressed in μg/mL and all experiments were done in triplicates in three individual experiments.

### Checkerboard assays

Synergy between grape pomace extract and 8 different antibiotics, as well as between grape pomace extract and 10 pure phenolic components identified therein were evaluated by the checkerboard method described by Motyl et al. [27] with minor modifications. Results of the combination between pure compounds and the whole extract should give a clue which component is responsible of the synergic effect against the multi-resistant clinical isolates and control strains. Briefly, eight serial, twofold dilutions of grape pomace extract and antibiotic were prepared. In a 96-well plate, 25 μL of each dilution of grape pomace extract was added in each vertical row, and 25 μL of antibiotic or pure compound dilution was added in each horizontal row. Both first horizontal and vertical rows were left with only one agent and the following rows contained a fixed amount of one agent and increasing concentrations of the second agent. In the selection of the range of concentrations, the MICs obtained for each tested agent and tested bacteria were considered. Grape pomace extract concentrations used ranged from 47 to 3,000 μg/mL and 3 to 3,000 μg/mL for antibiotic. To each well, 100 μL of MH broth and 10 μL bacterial suspension (1 x 10^8^ CFU/mL) were added. The plates were incubated at 37°C for 24 h and measured at 600 nm in an Elisa lector (Thermos Labsystems Multiskan FC Model). All tests were performed in triplicate in three different experiments. Fractional inhibitory concentrations (FIC) were calculated by the formula FIC_extract_ = (MIC extract + antibiotic / MIC extract) or FIC_antibiotic_ = (MIC extract + antibiotic / MIC antibiotic). The FIC index (FICI; [3]) for each combination was calculated by the sum of both FIC values, and results were interpreted as follows: FICI ≤ 0.5 synergic effect, 0.5 < FICI ≤ 4 additive effect and FICI ˃ 4 antagonistic effect [3]. These same formula were used for the calculation of the combinations of grape pomace extract with each phenolic compound.

### Cytotoxicity

In order to determine the cytotoxic potential of the synergistic combinations between grape pomace extract with antibiotics of different types, the cervical cancer cell line HeLa (ATCC CCL-2, USA) was used.

#### HeLa cell line culture

HeLa cells were grown in Dulbecco's modified medium (DMEM; Corning, USA) with bovine fetal serum 10% (FBS) supplemented with 100 U/mL penicillin, 100 μg/mL streptomycin (Corning Eagle, USA) in a modified Thermo Scientific air incubator (5% CO_2_ at 37°C). The medium was renewed every 2 days to reach 80% confluence, then the cells were transferred to T75 flasks, grown to reach 80% confluence, and finally the culture was divided into sterile 24-well plates.

#### Cytotoxicity evaluation

Approximately 10,000 cells per well in a 24-well plate were seeded in 100 μL of DMEM. The cells were treated with different concentrations of the grape pomace extract (750, 375, 188, 94 and 47 μg/mL, in 20 μL DMSO). Simultaneously, growth controls were performed, which consisted of cells incubated with culture medium alone and with 20 μL DMSO as solvent control. Finally, treated HeLa cells and the respective controls were incubated for 24 h in 5% CO_2_ at 37°C. All tests were carried out in triplicate. This same procedure was performed with the combinations between grape pomace extracts and the representatives of different classes of antibiotics that showed a synergistic effect at the minimal concentration. Concentrations used for ciprofloxacin and chloramphenicol were 20, 10, 5, 2.5, 1.25 and 0.62 μg/mL, for ampicillin were 750, 375, 188, 94, 47 and 23 μg/mL, while the concentration of grape pomace extract was 47 μg/mL, the lowest concentration that showed synergic effect. Cell viability was determined using propidium iodide as a marker of dead cells at a concentration of 1 μg/mL. Samples were analyzed on a FACSCanto II flow cytometer (Becton Dickinson). The analysis of the results was performed using the FACSDiva software.

### Statistical analysis

All data were analyzed using t-test using Graph Pad Prism 5 software.

## Results and discussion

### Minimum inhibitory concentration

Results of the susceptibility tests for *S*. *aureus* and *E*. *coli* strains are shown in Tables 1 and 2, respectively. The clinical isolates used in this study were resistant to more than three classes of antibiotics according to the criteria stablished by CLSI. This indicates that all isolates of both *S*. *aureus* and *E*. *coli* should be classified as multidrug resistant bacteria [28]. Even more, *S*. *aureus* isolates 8298–2 and 97–7 MRSA were resistant to all classes of antibiotics studied (fluoroquinolones, β-lactams, amphenicols and tetracyclines), while *E*. *coli* clinical isolates 16.1 and 33.1 showed the same trend. Three of the four *S*. *aureus* isolates and all *E*. *coli* were resistant to tetracycline. For chloramphenicol, three *S*. *aureus* and two *E*. *coli* isolates showed resistance to this drug. The minimum inhibitory concentrations (MIC) determined for each antibiotic by microdilution broth method are also shown in Tables 1 and 2. MIC values were high for all clinical isolates compared to the respective control strain. This tendency was similar for the MICs values obtained with the grape pomace extract, ranging between 1,500 to 3,000 μg/mL with the clinical isolates, compared to MIC values of 600 and 300 μg/mL obtained with the control strains *S*. *aureus* ATCC 6538 and *E*. *coli* ATCC 25922, respectively.

**Table 1:** Synergy analysis of grape pomace extract with different antibiotics against *S*. *aureus*.

			MIC (μg/mL)				
*S*. *aureus* strain	Antibiotic (Susceptibility)*		Antibiotic alone	Grape pomace extract alone	Antibiotic plus grape pomace extract	MIC reduction fold	FICI
**ATCC 6538**	Nalidixic acid	(R)	60	600	0.93	65	0.078
	Ciprofloxacin		1.5		0.02	75	0.028
	Norfloxacin		1.5		0.02	75	0.078
	Levofloxacin		1.5		0.02	75	0.078
	Oxacillin		3		0.05	64	0.047
	Tetracycline		1.5		0.05	30	0.094
	Chloramphenicol		16		0.25	64	0.047
**8275**	Nalidixic acid	(R)	300	1500	4.7	64	0.065
	Ciprofloxacin	(R)	30		0.9	32	0.062
	Norfloxacin	(R)	25		0.8	32	0.047
	Levofloxacin		10		0.3	33	0.047
	Oxacillin	(R)	50		1.6	31	0.063
	Tetracycline		5		0.08	63	0.047
	Chloramphenicol	(R)	75		2.3	33	0.062
**8298–2**	Nalidixic acid	(R)	200	1500	6.2	32	0.062
	Ciprofloxacin	(R)	150		4.7	32	0.063
	Norfloxacin	(R)	300		9.4	32	0.047
	Levofloxacin	(R)	20		0.31	65	0.031
	Oxacillin	(R)	300		4.7	64	0.031
	Tetracycline	(R)	8		0.25	32	0.047
	Chloramphenicol	(R)	150		4.7	32	0.047
**MRSA**	Nalidixic acid	(R)	300	3000	4,7	64	0.047
	Ciprofloxacin	(R)	15		0.5	30	0.063
	Norfloxacin	(R)	30		0.5	60	0.047
	Levofloxacin		3		0.1	30	0.061
	Oxacillin	(R)	150		2.3	65	0.047
	Tetracycline	(R)	750		23	33	0.062
	Chloramphenicol		1		0.02	50	0.056
**MRSA**	Nalidixic acid	(R)	150	1500	4.7	32	0.063
	Ciprofloxacin	(R)	50		1.6	31	0.063
	Norfloxacin	(R)	25		0.8	31	0.063
	Levofloxacin	(R)	8		0.25	32	0.063
	Oxacillin	(R)	300		4.6	65	0.047
	Tetracycline	(R)	500		7.8	64	0.047
	Chloramphenicol	(R)	5		0.16	31	0.063

*The susceptibility to antibiotics is indicated only if the bacterial strain is resistant (R).

**Table 2:** Synergy analysis of grape pomace extract with different antibiotics against *E*. *coli*.

			MIC (μg/mL)				
*E*. *coli* strain	Antibiotic (Susceptibility)*		Antibiotic alone	Grape pomace extract alone	Antibiotic plus grape pomace extract	MIC reduction fold	FICI
**ATCC 25922**	Nalidixic acid	(R)	16	300	0.25	64	0.078
	Ciprofloxacin		1		0.03	33	0.155
	Norfloxacin		1.5		0.045	33	0.155
	Levofloxacin		0.75		0.02	38	0.093
	Ampicillin	(R)	15		0.47	32	0.063
	Tetracycline		3		0.05	60	0.078
	Chloramphenicol		8		0.12	67	0.078
**16.1**	Nalidixic acid	(R)	2000	1500	31.2	64	0.047
	Ciprofloxacin	(R)	150		4.7	32	0.063
	Norfloxacin	(R)	200		3.1	65	0.031
	Levofloxacin	(R)	50		1.6	31	0.063
	Ampicillin	(R)	1500		47.0	32	0.063
	Tetracycline	(R)	100		3.1	32	0.047
	Chloramphenicol	(R)	10		0.3	33	0.062
**33.1**	Nalidixic acid	(R)	3000	3000	95.0	32	0.047
	Ciprofloxacin	(R)	20		1.25	16	0.078
	Norfloxacin	(R)	20		1.25	16	0.078
	Levofloxacin	(R)	30		0.47	64	0.047
	Ampicillin	(R)	1500		23.4	64	0.047
	Tetracycline	(R)	150		9.4	16	0.078
	Chloramphenicol	(R)	15		0.47	32	0.078
**A2UC**	Nalidixic acid	(R)	150	3000	4.7	32	0.047
	Ciprofloxacin	(R)	3		0.05	60	0.032
	Norfloxacin	(R)	6		0.2	30	0.063
	Levofloxacin		1.5		0.05	30	0.065
	Ampicillin	(R)	62		15	4	0.281
	Tetracycline	(R)	150		9.4	16	0.094
	Chloramphenicol		10		0.16	63	0.047

*The susceptibility to antibiotics is indicated only if the bacterial strain is resistant (R).

### Synergy interactions analysis

All combinations of grape pomace extract with the different classes of antibiotics were tested against each of the described clinical isolates of both *S*. *aureus* and *E*. *coli*. As shown in Table 1, against *S*. *aureus* isolates and the ATCC strain, a significant decrease of the MICs of all drugs was observed when the antibiotics were combined with the grape pomace extract reaching 30 to 75 times fold reduction, regardless if bacteria tested was or not resistant to the antibiotic.

FICI values obtained in the checkerboard assays were in the range of 0.03 to 0.094, indicating that all combinations studied have a synergistic effect (FICI ≤ 0.5) in all isolates, regardless of the mechanism of action of the tested antibiotic. Very similar results were observed in *E*. *coli* clinical isolates and the control strain (Table 2), in which the extract combinations with antibiotics (β-lactams, quinolones, tetracycline and chloramphenicol) showed reductions in the MIC of 4 to 67 times. Regarding FICI values, these ranged from 0.03 to 0.15, indicating that all tested combinations are synergistic. These results coincide with those reported in the literature in the sense that combinations of plant extracts with antibiotics belonging to different families show synergy against clinical isolates of *S*. *aureus* (MSSA, MRSA) and *E*. *coli*, significantly reducing the MIC of all antibiotics tested [8, 29]. *Camellia sinensis* extracts, particularly rich in polyphenols, when combined with oxacillin, decreased the MIC 256 times (from 256 μg/mL to 1 μg/mL) against MRSA strains [6]. Furthermore, this extract was able to potentiate the activity of levofloxacin *in vivo* against enterohemorrhagic *E*. *coli* O157 [30]. Our results indicate that the effect observed in all pomace extract—antibiotics combinations is independent of the mechanism of action of the antibiotic used, if the strain was resistant or not, and indifferent if bacteria is Gram-positive *S*. *aureus* or Gram-negative *E*. *coli*.

As grape is rich in phenolic compounds [11], their identification in the pomace extract should give a clue to determine which compound is responsible for the observed synergistic effect. Table 3 shows the list of 11 compounds identified in the extract with their relative abundance; 5 phenolic acids identified as gallic, syringic, vanillic, protocatechuic and p-coumaric, 5 flavonoids identified as quercetin, luteolin, kaempferol, (+)-catechin and (-)-epicatechin; and 1 tanin identified as ellagic acid. Different separation methods have been used for the identification of phenolic compounds in different *V*. *vinifera* varieties. Nicoletti et al. [31] analyzed nine grape varieties using RP-HPLC-MS and identified gallic acid, protocatechuic acid, catechin, epicatechin, rutin, among others. Sagdic et al. [32] determined the presence of 18 different phenolic compounds, including gallic acid, protocatechuic acid, p-coumaric acid, ferulic acid, vanillic acid, quercetin, kaempferol, (+)-catechin, (-)-epicatechin, hesperidin, among others, in 5 grape varieties cultivated in Greece. Similar results with different techniques based on HPLC were obtained by Kammerer et al. [33], Lafka et al. [34], Rockenbach et al. [9], and Mendoza et al. [12].

**Table 3:** Relative abundance of phenolic compounds in grape pomace extract.

Compounds	Retention time (min)	λ (nm)	Relative abundance (%)
Quercetin	52.1	255.5–369.3	26.3
Gallic acid	5.1	227.2–272.0	24.4
Protocatechuic acid	9.2	224.9–260.2	16.7
Luteolin	54.8	253.3–246.8	11.4
(+)-Catechin	13.5	228.4–279.1	3.7
(-)-Epicatechin	18.6	227.2–279.1	3.7
Vanillic acid	17.6	261.4–293.3	2.7
Kaempferol	53.8	266.1–365.7	2.4
Syringic acid	18.1	226.1–275.6	2.3
p-Coumaric acid	25.7	227.2–309.9	2.1
Ellagic acid	27.6	254.3–366.9	1.6

The relative abundance of the phenolics present in the grape pomace extract showed that gallic acid was the major component with a relative abundance of 26.3%, followed by protocatechuic acid, quercetin and luteolin (24.4, 16.7 and 11.4%, respectively; Table 3). The major components identified by Sadgic et al. [32] were gallic acid, chlorogenic acid, (+)-catechin, kaempferol, rutin and quercetin, results that are similar to those obtained in this work.

Considering the identification of phenolic compounds in the extract, checkerboard assays were performed between grape pomace extract and pure phenolic compounds to answer the question, which compound is responsible for the observed synergic effect, if the relative abundance is or not relevant, or the synergy effect is due to the presence of all components in the extract.

Tables 4 and 5 show the results obtained from the synergism analysis between grape pomace extract with each phenolic compound with *S*. *aureus* and *E*. *coli* strains, respectively. The MIC values of each of the phenolic compounds identified in the extract are high against both bacteria, including their respective control strains. For the pure phenolic acids (gallic, vanillic, syringic, p-coumaric and protocatechuic), the MIC ranged from 300 to 3,000 μg/mL for *S*. *aureus* and between 500 to 4,000 μg/mL for *E*. *coli*. The MICs obtained in the analysis of pure flavonoids (quercetin, luteolin, (-)-epicatechin and ellagic acid) ranged between 100 to 600 and 100 to 3,000 μg/mL for *S*. *aureus* and *E*. *coli*, respectively. In the case of (+)-catechin, MIC values were higher than 10,000 μg/mL in all isolates tested, indicating that it is inactive. Importantly, between control strains and the clinical isolates studied, no large differences were observed in the MICs for the identified phenolic compounds, either phenolic acids or flavonoids. Despite the high MIC values for the purified phenolic compounds, the combination of these compounds with the pomace extract reduced the MIC considerably, between 8 to 65 times for *S*. *aureus* and between 4 to125 times for *E*. *coli*.

**Table 4:** Synergy analysis between grape pomace extract and pure phenolic compounds in *S*. *aureus*.

		MIC (μg/mL)			
*S*. *aureus* strains	Phenolic compound	Phenolic compound alone	Phenolic compound plus grape pomace extract	MIC reduction fold	FICI
ATCC 6538	Gallic acid	1500	47	32	0.094
	Vanillic acid	1000	31.2	32	0.063
	Syringic acid	625	39	16	0.125
	p-Coumaric acid	1500	24	63	0.047
	Protocatechuic acid	750	23	33	0.093
	Ellagic acid	500	16	31	0.048
	Quercetin	375	11.7	32	0.063
	Luteolin	100	3.1	32	0.047
	(+)-Catechin	˃ 10000	-	-	-
	(-)-Epicatechin	500	62.5	8	0.162
8275	Gallic acid	1500	24	63	0.032
	Vanillic acid	1500	47	32	0.061
	Syringic acid	750	24	31	0.060
	p-Coumaric acid	300	9.4	32	0.060
	Protocatechuic acid	750	24	31	0.048
	Ellagic acid	250	15.6	16	0.094
	Quercetin	600	9.4	64	0.032
	Luteolin	500	31.2	16	0.094
	(+)-Catechin	˃ 10000	-	-	-
	(-)-Epicatechin	1000	15.6	64	0.047
8298–2	Gallic acid	3000	94	32	0.063
	Vanillic acid	1500	47	32	0.063
	Syringic acid	3000	47	64	0.047
	p-Coumaric acid	750	47	16	0.078
	Protocatechuic acid	750	24	31	0.063
	Ellagic acid	125	15.6	8	0.168
	Quercetin	150	4.7	32	0.063
	Luteolin	500	31.2	16	0.094
	(+)-Catechin	˃10000	-	-	-
	(-)-Epicatechin	1000	15.6	64	0.047
	Gallic acid	3000	47	64	0.047
	Vanillic acid	1500	47	32	0.063
	Syringic acid	1500	47	32	0.047
	p-Coumaric acid	1500	47	32	0.063
MRSA	Protocatechuic acid	750	47	16	0.094
622–4	Ellagic acid	250	3.9	64	0.031
	Quercetin	300	9.4	32	0.063
	Luteolin	500	7.8	64	0.031
	(+)-Catechin	˃10000	-	-	-
	(-)-Epicatechin	1000	15.6	64	0.031
	Gallic acid	2000	31	65	0.047
	Vanillic acid	1500	47	32	0.063
	Syringic acid	750	24	31	0.047
	p-Coumaric acid	750	24	31	0.047
MRSA	Protocatechuic acid	750	24	32	0.047
97–7	Ellagic acid	62.5	7.8	8	0.187
	Quercetin	100	3.1	32	0.062
	Luteolin	1000	62.5	16	0.093
	(+)-Catechin	˃10000	-	-	-
	(-)-Epicatechin	500	16	31	-0.063

**Table 5:** Synergy analysis between grape pomace extract and pure phenolic compounds in *E*. *coli*.

		MIC (μg/mL)			
*E*. *coli* strains	Phenolic compound	Phenolic compound alone	Phenolic compound plus grape pomace extract	MIC reduction fold	FICI
ATCC 25922	Gallic acid	2000	16	125	0.047
	Vanillic acid	750	23	32	0.089
	Syringic acid	1000	16	63	0.078
	p-Coumaric acid	750	47	16	0.125
	Protocatechuic acid	1000	16	63	0.078
	Ellagic acid	1000	62.5	16	0.094
	Quercetin	500	62.5	8	0.188
	Luteolin	200	25	8	0.156
	(+)-Catechin	˃10000	-	-	-
	(-)-Epicatechin	1000	125	8	0.188
16.1	Gallic acid	2000	62.5	32	0.063
	Vanillic acid	1000	62.5	16	0.125
	Syringic acid	500	62.5	8	0.141
	p-Coumaric acid	1000	62.5	16	0.125
	Protocatechuic acid	2000	62.5	32	0.063
	Ellagic acid	1000	250	4	0.281
	Quercetin	3000	375	8	0.156
	Luteolin	300	18.8	16	0.094
	(+)-Catechin	˃10000	-	-	-
	(-)-Epicatechin	3000	750	4	0.313
33.1	Gallic acid	2500	150	16	0.076
	Vanillic acid	4000	250	16	0.185
	Syringic acid	750	47	16	0.078
	p-Coumaric acid	1000	125	8	0.156
	Protocatechuic acid	4000	1000	4	0.500
	Ellagic acid	200	6.25	32	0.047
	Quercetin	500	15.6	32	0.062
	Luteolin	300	18.8	16	0.094
	(+)-Catechin	˃10000	-	-	-
	(-)-Epicatechin	3000	750	4	0.266
A2UC	Gallic acid	1500	94	16	0.078
	Vanillic acid	2000	62.5	32	0.047
	Syringic acid	1500	24	63	0.032
	p-Coumaric acid	2000	62.5	32	0.219
	Protocatechuic acid	2000	62.5	32	0.052
	Ellagic acid	500	31.2	16	0.156
	Quercetin	3000	188	16	0.078
	Luteolin	250	7.8	32	0.047
	(+)-Catechin	˃10000	-	-	-
	(-)-Epicatechin	5000	625	8	0.250

The fractional inhibitory concentration index (FICI) analysis by the checkerboard method showed for all combinations and bacterial isolates tested, values below 0.5, which is indicative of synergy (Tables 4 and 5). Results show that FICI values have no correlation with the relative abundance of the compounds in the extract (Table 3), since gallic acid, one of the most abundant compound identified presented FICI values between 0.032–0.094 for *S*. *aureus* and *E*. *coli*, similar range compared to the results of less abundant compounds like vanillic, syringic and p-coumaric acids, which FICI values range between 0.031 to 0.185 with all bacteria tested. These results suggest that it is not only one compound that is responsible for the observed synergistic effect, but that each of the identified compounds contributes to this effect to a greater or lesser extent (all combinations showed synergy), resulting in a multi-objective effect of grape pomace extract. This is strongly suggested by the synergy obtained when the extract was combined with antibiotics of different kinds, regardless of their mechanism of action.

Furthermore, it has been described that phenolic acids can break down the structure of the cytoplasmic membrane causing loss of integrity and eventual cell death [2, 35]. At sub-inhibitory concentrations, the compounds present in the extract would facilitate the entrance of the antibiotic to the cell cytoplasm, thus facilitating the entrance of fluoroquinolones, tetracycline and chloramphenicol, which have their site action within the bacterial cell, and less antibiotic dose would be needed. In this way, the multi-objective mechanism would be accomplished by disrupting the cytoplasmic membrane and some vital function as DNA replication, transcription or translation processes, depending on the antibiotic used.

Flavonoids has been described as causing cytoplasmic membrane damage, inhibition of peptidoglycan synthesis, nucleic acids synthesis inhibition, and/or energy transport inhibition [2]. Within the grape pomace extract, flavonoids luteolin, kaempferol, quercetin, (+)-catechin and (-)-epicatechin were identified. Cushnie and Lamb [36] determined the mechanism of action for (+)-catechin by cytoplasmic membrane damage, causing direct disruption of the lipid bilayers and alteration of the barrier function. Quercetin has also been shown that causes an increase in the permeability of the cytoplasmic membrane and dissipation of the membrane potential [22, 36]. Quercetin showed the highest relative abundance in our grape pomace extract. On the other hand, it has been described that flavonoids as corilagin and tellimagrandin I inhibit penicillin binding proteins (PBPs), specifically PBP2`(PBP2a) in methicillin resistant *S*. *aureus* (MRSA; [37]). This evidence support our results of synergy, compounds in grape pomace extract would interact with PBPs decreasing the effective concentration of β-lactamic antibiotics. Preliminary results obtained in our laboratory showed that grape pomace extract inhibits β-lactamases in *S*. *aureus* and *E*. *coli* (data not shown). It was also published that luteolin identified in grape pomace extract, increased the efficacy of different antibiotics, since it inhibits β-lactamase in MDR *E*. *coli* strains [21], affected the cytoplasmic membrane stability (possibly by generating hydrogen peroxide); inhibited enzymes involved in the synthesis of folic acid as dihydrofolate reductase [38]. These facts support a multi-target mechanism determined by the extract components. Therefore, the extract components could act at different targets increasing the susceptibility of bacteria and enhancing the activity of antibiotics, which result in significant reductions in the MIC of antibiotics.

However, it is important to highlight that even the grape has high concentration of phenolic compounds, it also contains other class of compounds like terpenes among which uvaol, β-amirin, palmitic acid, eicosanol, scualene and estearic acid were identified (data not shown). Antibacterial activity for these terpenes was also demonstrated [39, 40], suggesting that they could also contribute to the synergy effect.

### Cytotoxicity

**Fig 1:**
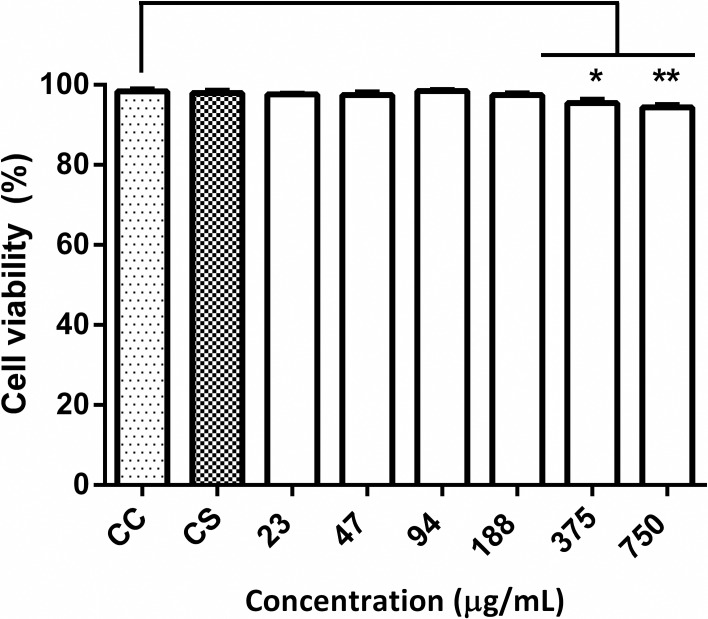
Viability of HeLa cells exposed to different concentrations of grape pomace extract. HeLa cells were incubated with different concentrations (μg/mL) of grape pomace extract and cell viability was determined after incubation with propidium iodide through efflux cytometry assay. As controls, HeLa cells were incubated without treatment (CC); with the solvent used with the extract (CS); *t-test *(P <0.002), ** (P <0.001) *v/s* CC.

The toxicity evaluation of grape pomace extract on HeLa cells is shown in Fig 1, indicating that the cell viability at low concentrations (23–188 μg/mL) is not affected compared to the control (98.3%) with viability percentages in a range of 98.4–97.4%. However, at higher concentrations (375 and 750 μg/mL), the viability was reduced to 95.4 and 94.4%, respectively. From these results, it can be concluded that the cytotoxicity of grape pomace extract increases with concentration. This result has been previously reported with plant extracts with high content of phenolic compounds [41, 42].

The same analysis was done with combinations of grape pomace extract with ciprofloxacin, chloramphenicol and ampicillin, using the minimum concentration of extract (47 μg/mL) in which a synergistic effect was observed. Concentrations used for the tested antibiotics are shown in Table 6. Antibiotics alone or combined with the grape pomace extract were non-toxic to HeLa cell line, at the concentrations tested and after 24 h incubation, showing no significant differences in the viability compared with the control. These results are promising for possible applications in animal models.

**Table 6:** Cytotoxicity on HeLa cells by antibiotics alone and in combination with grape pomace extract.

Cell viability (%)								
Ciprofloxacin			Chloramphenicol			Ampicillin		
Concentration (μg/mL)	Alone	With grape pomace extract*	Concentration (μg/mL)	Alone	With grape pomace extract*	Concentration(μg/mL)	Alone	With grape pomace extract*
0	98.9 ± 0.9	99.1 ± 0.5	0	98.6 ± 0.4	97.5 ± 1.1	0	98.3 ± 0.8	98.7 ± 0.6
0.62	97.2 ± 1.8	96.6 ± 2.1	0.62	97.1 ± 0.5	98.4 ± 0.6	23	98.1 ± 0.6	97.3 ±1.8
1.25	97.5 ± 0.8	97.4 ± 2.2	1.25	96.2 ± 0.4	96.2 ± 0.2	47	97.3 ± 0.7	95.7 ± 1.9
2.5	98.7 ± 1.4	98.6 ± 0.4	2.5	93.8 ± 1.4	95.7 ± 0.8	94	97.8 ± 0.9	96.6 ± 0.4
5	98.4 ± 0.9	98.6 ± 0.8	5	93.2 ± 1.4	96.4 ± 0.1	188	97.6 ± 0.4	98.4 ± 0.4
10	97.6 ± 1.3	98.8 ± 1.3	10	95.4 ± 1.1	96.7 ± 0.5	375	97.4 ± 0.7	97.8 ± 0.5
20	97.0 ± 2.3	97.9 ± 1.1	20	96.5 ± 1.1	98.3 ± 0.9	750	96.7 ± 1.1	97.4 ± 0.8

*The concentration of the grape pomace extract was 47 μg/mL; t-test P ˃ 0.05.

## Conclusions

Grape pomace extract obtained from Cabernet sauvignon variety, used in combination with antibiotics of different classes against *S*. *aureus* and *E*. *coli* strains, especially multi-drug resistant clinical isolates, showed synergy reducing significantly the MICs of different classes of antibiotics studied in this work. This synergistic effect may be due to the joint action of the compounds contained in the extract, and not to a particular compound. Moreover, pomace extract–antibiotic combinations are not toxic for the HeLa cell line at concentrations in which the synergistic effect was determined. Therefore, these mixtures are good candidates for animal model testing in order to enhance the effect of antibiotics of different classes and thus restore the currently unused agents due to the phenomenon of resistance. Furthermore, the use of grape pomace is a good alternative for this purpose as being a residue of the wine industry, so that extracts and/or phenolic compounds could be obtained from this waste at low cost.
